# Relationship of Spontaneous Retinal Vein Pulsation with Ocular Circulatory Cycle

**DOI:** 10.1371/journal.pone.0097943

**Published:** 2014-05-20

**Authors:** Mijin Kim, Eun Ji Lee, Je Hyun Seo, Tae-Woo Kim

**Affiliations:** 1 Department of Ophthalmology, Konyang University, Kim's Eye Hospital, Myung-Gok Eye Research Institute, Seoul, Korea; 2 Department of Ophthalmology, Seoul National University College of Medicine, Seoul National University Bundang Hospital, Seongnam, Korea; 3 Department of Ophthalmology, Pusan National University Yangsan Hospital, Pusan, Korea; Bascom Palmer Eye Institute, University of Miami School of Medicine, United States of America

## Abstract

**Purpose:**

To determine the timing of spontaneous venous pulsation (SVP) relative to the ocular circulatory cycle by using the movie tool of confocal scanning laser ophthalmoloscope.

**Methods:**

A video recording of the fundus was obtained using a confocal scanning laser ophthalmoscope (Spectralis HRA, Heidelberg Engineering, Heidelberg, Germany) at 8 frames/s in 47 eyes (15 glaucoma patients and 32 glaucoma suspects) with visible pulsation of both the central retinal artery (CRA) and vein (CRV). The timing of the maximum and minimum diameters of the CRA (CRA_max_ and CRA_min_, respectively) and CRV (CRV_max_ and CRV_min_, respectively) was identified during four pulse cycles. The interval between CRV_min_ and CRA_min_, and between CRV_max_ and CRA_max_ was expressed as the number of frames and as a percentage of the ocular circulatory cycle.

**Results:**

The ocular circulatory cycle (from one CRA_max_ to the next) lasted 7.7±1.0 frames (958.8±127.2 ms, mean±SD), with a mean pulse rate of 62.6 beats/min. The diameter of the CRA was increased for 2.4±0.5 frames (301.9±58.8 ms) and decreased for 5.3±0.9 frames (656.9±113.5 ms). CRV_max_ occurred 1.0±0.2 frames after CRA_max_ (equivalent to 13.0% of the ocular circulatory cycle), while CRV_min_ occurred 1.1±0.4 frames after CRA_min_ (equivalent to 14.6% of the ocular circulatory cycle).

**Conclusions:**

During SVP, the diameter of the CRV began to decrease at early diastole, and the reduction persisted until early systole. This finding supports that CRV collapse occurs during ocular diastole.

## Introduction

Spontaneous retinal pulsation (SVP) manifests as a rhythmic variation in the caliber of the retinal vein near to or on the optic disc, and is visible in approximately 80–90% of healthy individuals [1]–[3]. SVP is significantly less common in glaucoma patients than in glaucoma suspects [1], [4], [5]. It is generally considered that the absence of SVP in glaucoma is likely due to increased resistance of the retrolaminar central retinal vein (CRV) [1], [6]–[9].Thus, the frequent absence of SVP in glaucoma patients may support that vascular factors play a role in the pathogenesis of glaucomatous optic neuropathy [4]. However, the precise pathogenetic relationship between the absence of SVP and optic nerve damage remains to be elucidated. To investigate such relationship, it is fundamental to understand the physiology of SVP.

When SVP was first described by Coccius [10], it was considered that the venous collapse was induced by an elevation in intraocular pressure (IOP) secondary to blood influx to the eye during systole. Since then, venous collapse has been considered to be caused by the IOP higher than the pressure in the retrolaminar CRV during systole [11], [12]. Later, Levine [13] questioned this concept based on the findings that the retinal venous pressure is always higher than the IOP [14]–[16], and that the IOP is instantly transmitted to the retinal veins [15]. He suggested that the greater fluctuation of IOP compared to that of the retrolaminar venous pressure plays a major role in SVP [13]. This theory is also based on the assumption that the venous collapse occurs at ocular systole.

However, Kain et al. [17] recently suggested that the venous collapse associated with SVP occurs at ocular diastole rather than systole. They based this suggestion on their observation of the relationship between the IOP fluctuation (as assessed by Goldmann applanation mires) and retinal vein diameter. They found that the minimum and maximum vein diameters occurred shortly after the minimum and maximum IOPs had been reached, respectively, indicating that venous collapse occurs in synchrony with ocular diastole. However, one limitation of their study was that the IOP fluctuation and fundus were not recorded simultaneously, and only ten patients were included. The novelty and this limitation of their study justify further investigation of this issue.

Our group recently demonstrated that the movie tool of confocal scanning laser ophthalmoscope (Spectralis HRA, Heidelberg Engineering, Heidelberg, Germany) may be useful for recording SVP [4]. The purpose of the present study was to determine the timing of SVP in relation to the central retinal artery (CRA) pulse using real-time fundus video imaging with the Spectralis HRA system in a relatively large number of patients. This approach allows a synchronized analysis of the timing of CRV collapse relative to the ocular circulatory cycle.

To the best of our knowledge, this is the first study to evaluate the phase relationship between the CRA pulse and CRV pulse.

## Materials and Methods

This study was a retrospective review of fundus video clips of the optic disc, which were collected for our previous study [4]. This study was approved by the Seoul National University Bundang Hospital Institutional Review Board. Written informed consent to participate was obtained from all subjects prior to recording the fundus video, and the study followed the tenets of the Declaration of Helsinki.

Before the study, each subject received the following comprehensive ophthalmic examinations: visual acuity measurement, Goldmann applanation tonometry, refraction tests, slit-lamp biomicroscopy, gonioscopy, dilated stereoscopic examination of the optic disc, disc photographs (EOS D60 digital camera, Canon, Utsunomiyashi, Tochigiken, Japan), retinal nerve fiber layer (RNFL) thickness measurement, and real-time fundus video recording using spectral-domain optical coherence tomography (SD-OCT; Heidelberg Engineering), and standard automated perimetry (Humphrey Field Analyzer II 750, 24-2 Swedish interactive threshold algorithm, Carl Zeiss Meditec).

A glaucoma suspect was defined as having elevated IOP or a suspicious-appearing optic disc but a normal visual field. Primary open-angle glaucoma (POAG) was defined as having characteristics of glaucomatous optic neuropathy, such as rim thinning, notching, and RNFL defect, a glaucomatous visual field defect, and an open iridocorneal angle. Glaucomatous visual field defect was defined as (1) outside normal limit on glaucoma hemifield test or (2) 3 abnormal points, with P<5% probability of being normal, one with P<1% by pattern deviation; or (3) pattern standard deviation of <5% confirmed on 2 consecutive tests. Only reliable visual field tests were included in the analysis.

To be included in our previous study, subjects were required to have at least two IOP measurements before receiving antiglaucoma treatment, a best-corrected visual acuity of ≥20/40, a spherical equivalent refractive error range from −7.0 to +3.0 diopters (D), cylinder correction of >±3.0 D, an open anterior chamber angle, and reliable visual fields (a fixation loss rate of ≤20%, and false-positive and false-negative error rates of ≤25%). Subjects with a history of ocular surgery other than uncomplicated cataract surgery, history of ocular trauma and uveitis, or other diseases affecting the visual field (e.g., diabetic retinopathy, retinal vein occlusion, and ischemic optic neuropathy) were excluded. Subjects with poor quality of movie recording of the fundus (media opacity and severe eye movement) were also excluded.

Of the subjects included in the previous study, only subjects who had pulsation of both CRA and CRV markedly visible on fundus movie recording were selected and included in the present study. In cases in which both eyes of a subject were eligible for the study, only one randomly selected eye was included.

### Observation of SVP

SVP was observed using fundus video recordings that were acquired with a confocal scanning laser ophthalmoscope (Spectralis HRA) in near-infrared mode (820 nm). Real-time fundus video clips centered on the optic nerve head of each eye were recorded by a skilled technician after inducing pupil dilation. The video clips were recorded at a frame rate of 8 frames/s for a total of 20s each, and had a resolution of 768 by 768 pixels. The video clips were reviewed after adjustment of image movement using an eye-movement correction tool that was installed in the device, and eligible subjects (i.e., those with markedly noticeable arterial and venous pulsation) were selected by a glaucoma specialist (M.K.). The video clips of the selected patients were then reviewed in slow motion (1 frame/2 s) by two glaucoma specialists (M.K. and E.J.L.), who independently evaluated the sequential changes in the diameter of the central retinal vessels, and identified the timing of the maximum and minimum diameters of both the CRA (CRA_max_ and CRA_min_, respectively) and CRV (CRV_max_ and CRV_min_, respectively; see Video Clips S1 and S2). In cases of disagreement in the judgments of vessel pulsation movement, a third glaucoma expert (T-W.K.) served as an adjudicator. The frame intervals between CRA_max_ and CRV_max_, and between CRA_min_ and CRV_min_ are presented as percentages of the ocular circulatory cycle (i.e., the total number of frames between one CRA_min_ and the next). Images from four pulse cycles were evaluated for each patient, and the measurements obtained from these four cycles were averaged and used for the analysis.

Interobserver agreement for determining the constriction/dilatation of vessels in two successive frames was assessed using the kappa statistic in 20 randomly selected frames. The strength of agreement was categorized as following in accordance with the method proposed by Landis and Koch [18]: 0  =  poor; 0–0.20  =  slight; 0.21–0.40  =  fair; 0.41–0.60  =  moderate; 0.61–0.80  =  substantial, and 0.81–1.00  =  almost perfect. The interobserver reproducibility of the timing of the minimum and maximum diameters of the vessels was measured by two observers, and the intraclass correlation coefficients (ICCs) and their confidence intervals (CIs) were calculated. Except where indicated otherwise, the data are presented as mean±SD values.

## Results

Of the 434 patients included in our previous study [4], 48 (32 glaucoma suspects and 16 POAG patients) had marked pulsation in both the CRA and CRV that was observable in the fundus video recordings. Of these, 1 glaucoma -patient was excluded due to a highly variable ocular circulatory cycle that was suggestive of cardiac arrhythmia, leaving a sample of 47 patients. Twenty-one (44.7%) of the subjects were men and 26 were women, and they were aged 60.8±12.3 years and had a refractive error of−0.02±1.95 D (range, +3.25 to −6.13 D). The IOP at the time of SD-OCT was 14.8±4.1 mmHg, and the visual field mean deviation was −1.43±2.77 dB (range, +0.85 to −12.44 dB; Table 1).

**Table 1:** Demographics of the subjects.

Variable	Value (*n* = 47)
**Age (years)**	60.8±12.3
**Males, ** ***n*** ** (%)**	21 (44.7%)
**IOP at imaging (mmHg)**	14.8±4.1
**Refractive errors (D)**	−0.02±1.95
**Visual field mean deviation (dB)**	−1.43±2.77

IOP, intraocular pressure

There was almost perfect interobserver agreement for the constriction/dilatation of the CRA and CRV (kappa  =  0.836 and 0.910, respectively). The interobserver ICCs for measurement of the timing of the minimum and maximum diameters of the vessels are given in Table 2.

**Table 2:** Interobserver intraclass correlation coefficients (ICCs) for measurements of the timing of the minimum and maximum diameter of the vessels.

	ICC	95% confidence interval
**Timing of CRA_max_**	0.955	0.921–0.975
**Timing of CRA_min_**	0.970	0.947–0.983
**Timing of CRV_max_**	0.908	0.840–0.948
**Timing of CRA_min_**	0.940	0.894–0.966

CRA_max_, maximum diameter of the central retinal artery; CRV_max_, maximum diameter of the central retinal vein; CRA_min_, minimum diameter of the central retinal artery.

**Figure 1:**
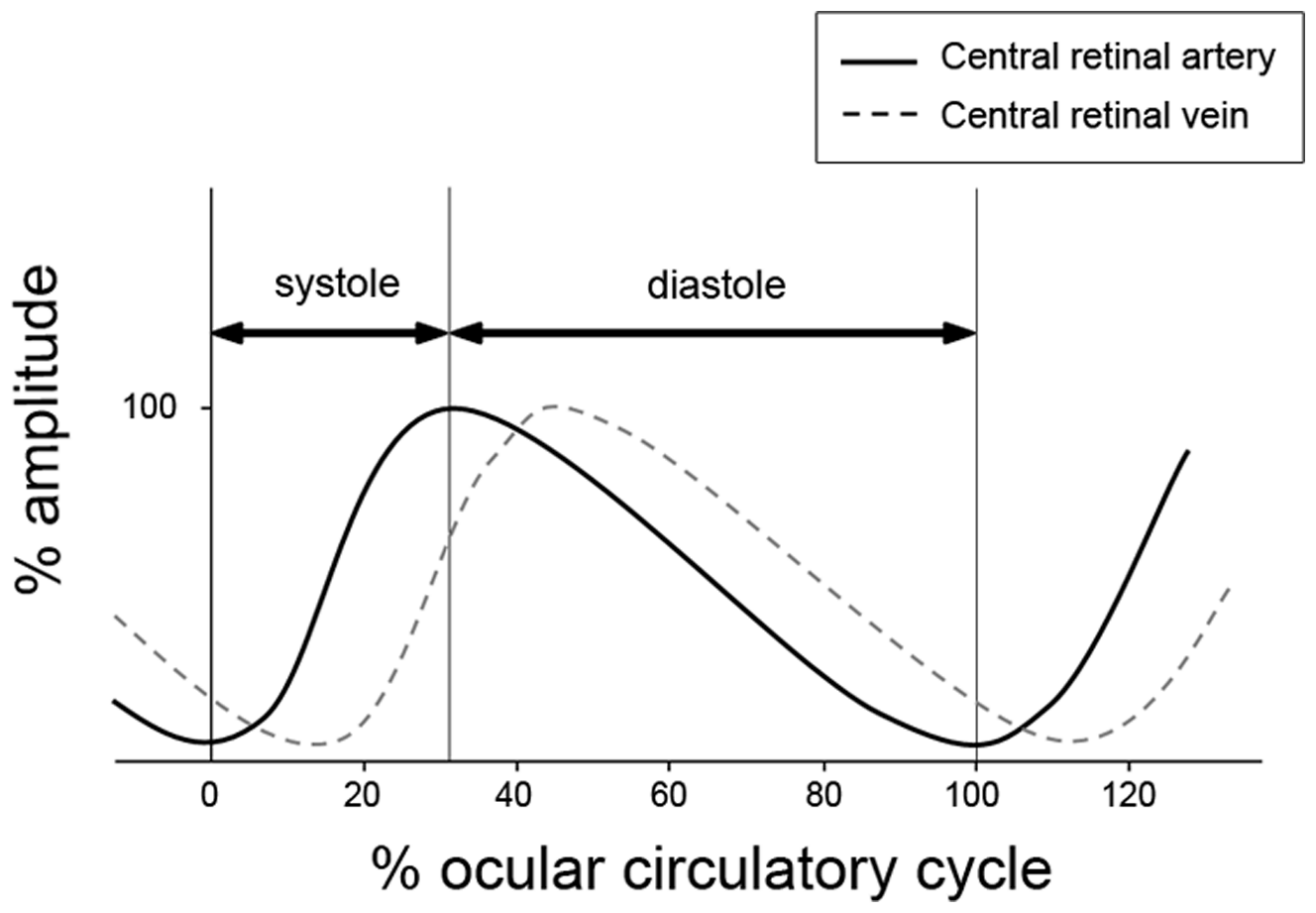
Schematic diagram illustrating the central retinal artery and vein pulse curves during one ocular circulatory cycle. The amplitude is presented in order to facilitate the understanding of the pulsation cycle, although the amplitude is not based on the actual measurement of the diameters of the artery and vein.

The ocular circulatory cycle (interval between one CRA_min_ and the next) was 7.7±1.0 frames, which was equivalent to a duration of 958.8±127.2 ms, with a mean pulse rate of 62.6 beats/min. Ocular systole lasted 2.4±0.5 frames (31.3% of the ocular circulatory cycle), while ocular diastole lasted 5.3±0.9 frames (68.7% of the ocular circulatory cycle). CRV_min_ occurred after 1.0±0.2 frames (equivalent to 13.0% of the ocular circulatory cycle; 95% CI, 12.2–13.8% of the ocular circulatory cycle). CRV_max_ occurred after 1.1±0.4 frames (equivalent to 14.6% of the ocular circulatory cycle; 95% CI, 13.1–16.1% of the ocular circulatory cycle; Figure 1).

**Figure 2:**
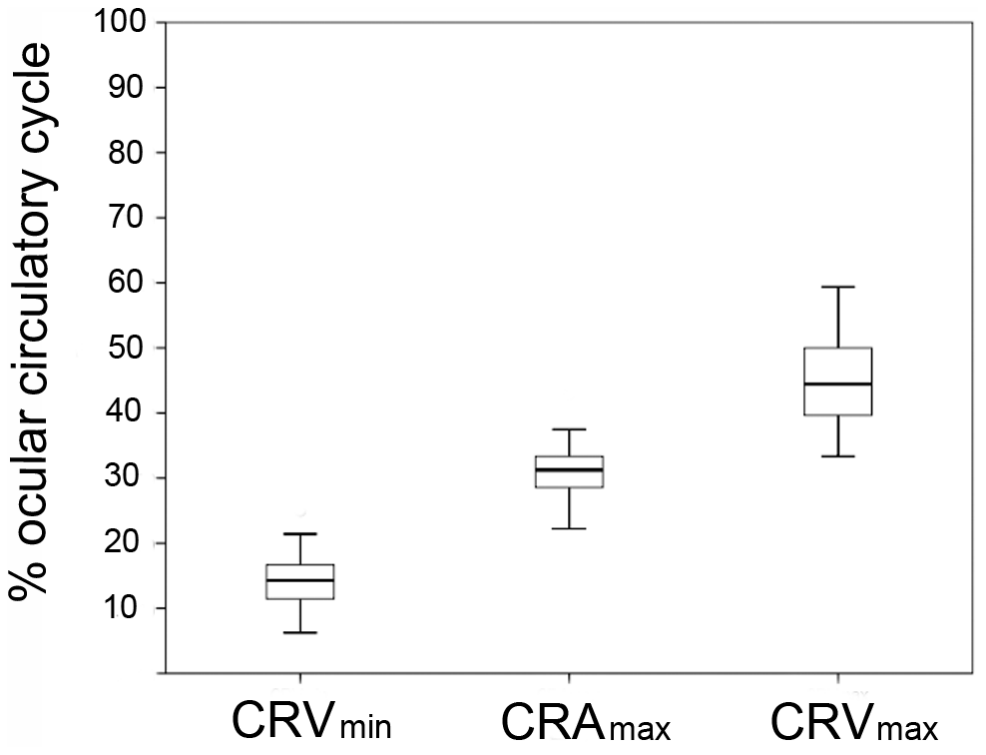
Box plots showing the distributions of CRA_max_, CRV_min_, and CRV_max_ expressed as percentages of the ocular circulatory cycle relative to CRA_min_. The time point of CRA_min_ was set as the reference (i.e., time zero). CRA_max_, maximum diameter of the central retinal artery; CRA_min_, minimum diameter of the central retinal artery; CRV_max_, maximum diameter of the central retinal vein; CRV_min_, minimum diameter of the central retinal vein.

Figure 2 shows the distributions of CRA_max_, CRV_min_, and CRV_max_ expressed as percentages of the ocular circulatory cycle relative to CRA_min_, with the time point of CRA_min_ set as the reference (i.e., time zero). CRV_min_, CRA_max,_ and CRV_max_ were observed at 14.8±5.1%, 31.3±5.4%, and 44.7±6.6% of the ocular circulatory cycle, respectively.

**Figure 3:**
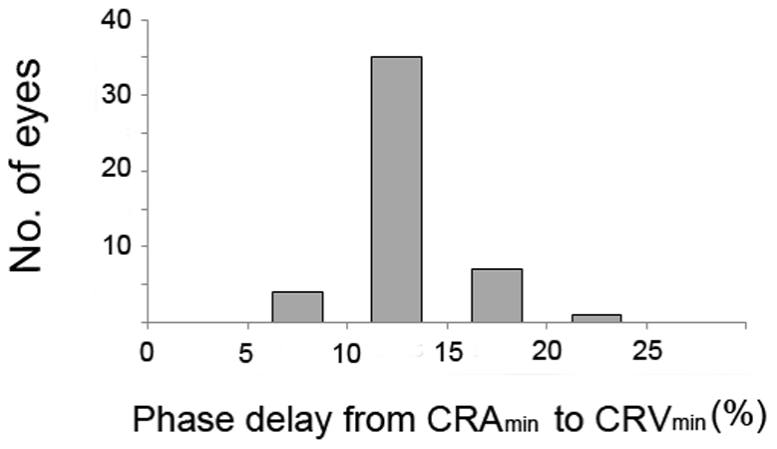
Frequency distribution of the phase delay between CRA_min_ and CRV_min_, expressed as a percentage of the ocular circulatory cycle. CRA_min_, minimum diameter of central retinal artery; CRV_min_, minimum diameter of central retinal vein.

**Figure 4:**
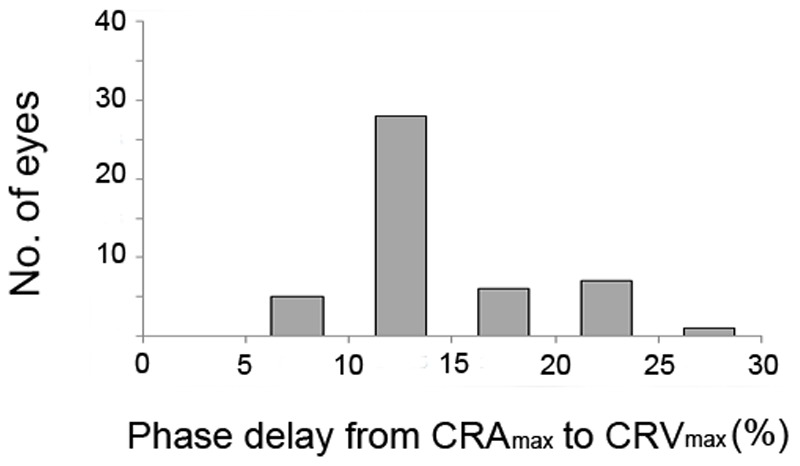
Frequency distribution of the phase delay between CRA_max_ and CRV_max_, expressed as a percentage of the ocular circulatory cycle. CRA_max_, maximum diameter of central retinal artery; CRV_max_, maximum diameter of central retinal vein.

Figures 3 and 4 present the frequency distributions of the CRV_min_ and CRV_max_ delays as percentages of the ocular circulatory cycle. In all patients, CRV_min_ occurred after CRA_min_ by at least 9% of the ocular circulatory cycle. Most of the patients (83.0%) exhibited a phase delay of 9–15% of the ocular circulatory cycle between CRA_min_ and CRV_min_ (Figure 3). CRV_max_ also occurred after CRA_max_ in all patients by at least 6% of the ocular circulatory cycle (Figure 4). The phase delay from CRA_min_ to CRV_min_ (*P* = 0.518) and from CRA_max_ to CRV_max_ (*P* = 0.257) was not significantly correlated with the IOP at the time of disc scanning.

## Discussion

Since the first documentation of SVP by Coccius [10] it has been considered that the CRV collapses during systole. However, this understanding was recently challenged by Kain et al. [17] and Morgan et al. [19], who demonstrated that CRV collapse occurred at ocular diastole. Their study nicely posited a question related to the long-held belief that CRV collapses at ocular systole. The current study revisited this issue by recruiting a larger sample, and using a different approach. It was found that CRV_max_ occurred after CRA_max_ (peak systole) and CRV_min_ occurred after CRA_min_ (peak diastole). These findings suggest that the CRV diameter started to decrease at early diastole and reached a minimum at early systole, which is consistent with the findings of previous studies. [17], [19].

When fluid drains from a collapsible vessel within a pressurized chamber to the external space, the walls of the collapsible vessel will oscillate within the chamber near the exit region [20]–[22]. It is known that the collapse of the vessel will be synchronized with the most negative transmural pressure induced in the vessels near the exit region [23]. For the CRV, the eye IOP is analogous to the pressurized chamber, and the cerebrospinal fluid is analogous to the external space. Along with drainage of the blood from the eye to the retrobulbar tissue, the CRV would oscillate near the lamina cribrosa (i.e., the exit region). In light of these dynamics, our data together with those of Kain et al. [17] and the observations of Morgan et al. [19] suggest that the negative transmural pressure induced in the CRV is initiated in early diastole.

Several hypotheses have been proposed for the mechanism by which venous collapse occurs during diastole. The first hypothesis is based on the time difference between the reductions in ICP and IOP, both of which occur during cardiac diastole [19], [24]. According to the characteristic relationship between IOP and ICP, the reduction in ICP tends to occur before that in IOP in early diastole [19], [25]–[27]. This may result in a negative transmural pressure within the CRV during diastole, leading its collapse during this period. Another explanation for this phenomenon is based on the difference in amplitude between ICP and IOP. This hypothesis claims that the ICP pulse has a larger amplitude than the IOP pulse, which would lead to an increased pressure drop between the two compartments during diastole [17]. However, until now there has been no published evidence to confirm that the ICP amplitude is greater than the IOP amplitude.

After collapse of the CRV, ICP increases more rapidly than the IOP in systole according to Morgan et al. 's study [19], leading to intraocular blood accumulation (i.e., CRV dilation) during systole. Thus, ICP and IOP have phase difference during the whole pulse cycle. Such phase difference between the ICP and IOP may have physiological advantages. First, the earlier ICP drop than the IOP drop during diastole may favor a more rapid drainage of blood from the intraocular compartment toward the retrobulbar space [19]. Second, the phase difference between ICP and IOP would generate a period when the pressure difference between the two is reduced (or potentially reversed). The existence of such a period may help the retrograde axoplasmic flow within the optic nerve to enter the eye [28], [29]. It is reasonable to assume that such a period exists during the period of CRV filling, whereby the increase in ICP impedes the downstream blood flow. According to the present study, CRV filling occurred during 32.9% of the pulse cycle (from CRV_min_ to CRV_max_), suggesting that there is a considerable time window during which the retrograde axoplasmic flow could be facilitated.

Our study has limitations. First, the fundus video was recorded at only 8 frames/s, which was the maximum frame rate available when using the Spectralis movie tool. However, the limitation of a large interframe interval (0.125 s) was partly overcome by averaging the data from four cycles. Nonetheless, this limits the precise calculation of the interval between CRV_min_ and CRA_min_, and between CRV_max_ and CRA_max_. Thus, the present results may be of value only for determining the temporal relationship between the CRV and CRA pulses, and not for calculating the exact timing of the phase difference between the two. Second, only subjects who had marked observable pulsation of both the artery and vein in the fundus video recording were included. Thus, the findings may not be applicable to the general population. Some of the excluded patients might have had oscillatory blood flow in the artery and vein, although this was not clearly noticeable. It is possible that those subjects had different pulsation timing characteristics.

In conclusion, the CRV collapse associated with SVP started at ocular diastole and lasted until early systole. This observation supports the recently discovered notion that CRV collapse occurs during ocular diastole.

## Supporting Information

Video S1 **Video clip of the fundus of the right eye of a 64-year-old woman.** Note the change in the diameter of the inferotemporal artery (white arrowhead). The diameter of the indicated artery gradually decreases from frame 1 to frame 3, and then increases abruptly in frame 4, suggesting that CRA_min_ occurred between frames 3 and 4. The diameter of the artery gradually increases in frames 4 and 5, and then decreases from frame 6, suggesting that CRA_max_ occurred between frames 5 and 6. CRA_min_, minimum diameter of the central retinal artery; CRA_max_, maximum diameter of the central retinal artery.
(AVI)

Video S2 **The same video recording as shown in video clip 1.** Note the change in the diameter of the inferior hemiretinal vein (black arrowhead). The diameter of the indicated vein gradually decreases between frames 1 and 4, and then increases abruptly in frame 5, suggesting that CRV_min_ occurred between frames 4 and 5. The diameter of the vein gradually increases in frames 5 and 6, and then decreases from frame 7, suggesting that CRV_max_ occurred between frames 6 and 7. CRA_min_, minmum diameter of the central retinal artery; CRA_max_, maximum diameter of the central retinal artery.
(AVI)
